# Epidemiological, Clinical, and Biological Characteristics of Infantile Visceral Leishmaniasis: A Study of 69 Cases

**DOI:** 10.7759/cureus.82851

**Published:** 2025-04-23

**Authors:** Smail Ghouzraf, Aicha Bourrahouat, Mohamed Zouhri, El Mustapha El Mezouari, Redouane Moutaj

**Affiliations:** 1 Parasitology and Mycology, Faculty of Medicine and Pharmacy, Avicenna Military Hospital, Marrakesh, MAR; 2 Pediatrics, Faculty of Medicine and Pharmacy, Mohammed VI University, Marrakesh, MAR

**Keywords:** clinics, epidemiology, indirect diagnosis, infantile visceral leishmaniasis, leishmania infantum, marrakesh, morocco, phlebotomus

## Abstract

Visceral infantile leishmaniasis is a parasitic infection caused by the multiplication in the reticuloendothelial system of a flagellate protozoan of the Leishmania genus, specifically the infantum species in the Moroccan context. It is transmitted through the bite of an insect known as a sandfly (phlebotomine). This study aimed to outline the epidemiological, clinical, and biological profiles of this pathology in a pediatric setting and to examine the different therapeutic modalities in order to improve its prognosis. A retrospective study spread over a period of seven years (from January 1, 2017, to December 31, 2023) was carried out in the Parasitology Laboratory of the Avicenna Military Hospital and the Pediatric Department of Mohammed VI University Hospital Center. For the 69 patients included in this study, we collected geographical origin, age, sex, clinical data, and biological data. The average age was two years and 11 months with a slight masculine predominance. The clinical triad - fever, pallor, and splenomegaly - was found in 53 (78%) patients. Normochromic normocytic anemia was almost constant (n=67, 97%) followed by thrombopenia (n=60, 87%) and leukopenia (n=40, 57%). The diagnosis has been confirmed by the search for the parasite in the bone marrow in 61 (89%) cases. The indirect diagnosis was made using screening techniques, such as ELISA, IFI, and rapid diagnostic tests, associated with the western blot confirmation technique. These techniques made it possible to recover the diagnosis in the remaining eight (11%) cases. N-methylglucamine was used as a first-intention treatment. A favorable evolution was observed in the majority of patients; however, we deplored three deaths and two relapses which pose a real management challenge, requiring collaboration between the clinician and the parasitologist for better case analysis.

## Introduction

Visceral leishmaniasis (VL) is a systemic vector-borne parasitic disease caused by intracellular protozoa of the genus Leishmania (L) and transmitted by the bite of an infected female Phlebotomine sand fly of the genus Phlebotomus. VL is a major and serious public health problem in 62 countries; more than 90% of global VL cases occur in just six countries - India, Bangladesh, Sudan, South Sudan, Brazil, and Ethiopia [1]. The transmission characteristics of VL differ in different geographical regions; in the Mediterranean basin, Brazil, and parts of Africa, the dog is the main reservoir and VL is zoonotic; while in the Indian subcontinent and parts of Africa, it is anthroponotic [2].

The first case of human visceral leishmaniasis in Morocco was mentioned by Klippel and Monier-Vignard in 1922 [3]. Observations recorded showed a wide dispersion of the disease in various provinces of the country. The northern regions are the most frequently cited, such as Tangiers, Fez, Casablanca, and Azrou, other publications are located further south, such as Irherm, Tata, and Goulimime [4].

The most often isolated parasite is *Leishmania infantum *zymodeme MON-1, having dogs as the animal reservoir; and as vectors, *Phlebotomus perniciosus *and *Phlebotomus ariasi *[5]. Therefore, VL has been classified since 1995 as a notifiable disease and in order to eradicate this pathology, a control program has been established.

In its typical form, infantile visceral leishmaniasis (IVL) is suspected in front of the classic triad - anemia, anarchic fever, and splenomegaly. The diagnosis of certainty is based on the demonstration of Leishmania bodies in the bone marrow, or the detection of antibodies by serological assays. The biological diagnosis is of utmost importance because the clinical features are not specific and can be easily mistaken for other febrile illnesses. Reliable laboratory methods are mandatory for accurate diagnosis. Early case detection followed by adequate treatment is central to the control of VL. This study aimed to outline the epidemiological, clinical, and biological profiles of this pathology in a pediatric setting and to examine the different therapeutic modalities to improve its prognosis.

## Materials and methods

Study population

This is an seven-year descriptive retrospective study conducted from January 1, 2017, to December 31, 2023. We included children hospitalized for suspicion of visceral leishmaniasis in the pediatric department of Mohammed VI University Hospital and in whom the confirmation of the diagnosis was made in the Parasitology laboratory at the Avicenna Military Hospital (AMH) of Marrakesh.

Inclusion and Exclusion Criteria

Any patient hospitalized in the pediatrics department with a clinical presentation suggestive of visceral leishmaniasis, having Leishmania bodies in the bone marrow, and/or a positive serology for leishmaniasis. Visceral leishmaniasis in children treated in Marrakesh. Records that were deemed unusable, or where the diagnosis was not confirmed, were excluded. We also excluded children with VL who were not registered during the study period and records confirmed outside the Avicenna Military Hospital (AMH) parasitology laboratory.

Biological parameters

For the purpose of this study, we exclusively included patients who had a confirmed diagnosis of IVL through the presence of amastigote forms of leishmaniasis bodies in the bone marrow (n=62), as well as those who exhibited clinical symptoms suggestive of IVL and had a positive serology (n=7). It should be noted that eight patients had both a positive direct diagnosis and serology.

Direct Diagnosis

The direct examination of the bone marrow represents the means of reference and first intention for the diagnosis of visceral leishmaniasis. The bone marrow harvest is obtained by a puncture of the iliac crest in most cases and is spread on slides in a thin smear. The sample is received in the laboratory and stained with May-Grünwald Giemsa (MGG) or by rapid staining kits such as RAL 555 (Lund, Sweden: CellaVision).

Indirect Diagnosis

The detection of the antibodies was carried out by different techniques such as ELISA, indirect immunofluorescence (IFI), and rapid diagnostic test - IT LEISH, while the serological confirmation was carried out by the immunoblot technique - western blot.

Clinical parameters

For each patient, an information sheet containing the epidemiological (age, sex, geographical origin), clinical (first appointment date, consultation times, symptomatology), and paraclinical (biological and radiological analyses) therapeutic data were used.

Statistics

Data were entered using Excel 2021 software (Redmond, WA: Microsoft Corp.). A descriptive analysis of the epidemiological, clinical, and biological characteristics of visceral leishmaniasis in the pediatric setting was conducted. The results were presented in the form of graphs and tables. Statistical analysis was carried out using SPSS version 21 (Armonk, NY: IBM Corp.). A univariate analysis was performed using the Student's t-test and Fisher's exact test, with a significance threshold of 5% (p<0.05).

Ethics statement

According to the ethical approval of this retrospective study, all samples were anonymized. For the hospital survey, the data were extracted from patient records by the clinician in charge and transferred to an individual questionnaire, without including any personal data that could identify individual patients.

## Results

A total of 69 children were hospitalized for confirmed visceral leishmaniasis. The average age of patients was two years and 11 months (extremes of four months to 10 years). Infants and toddlers were the most affected by the disease, with 32 (46.16%) of all cases falling within the age group of four months to two years, followed by 24 (34.65%) in the two to four years group. There was a slight male predominance, with a sex ratio of 1.15 (37 boys to 32 girls). Half of the patients in this study came from the Drâa-Tafilalet region, accounting for 36 (51.81%), and 23 (33.18%) from the Marrakesh-Safi region. Most patients came from low-income rural environments.

**Figure 1:**
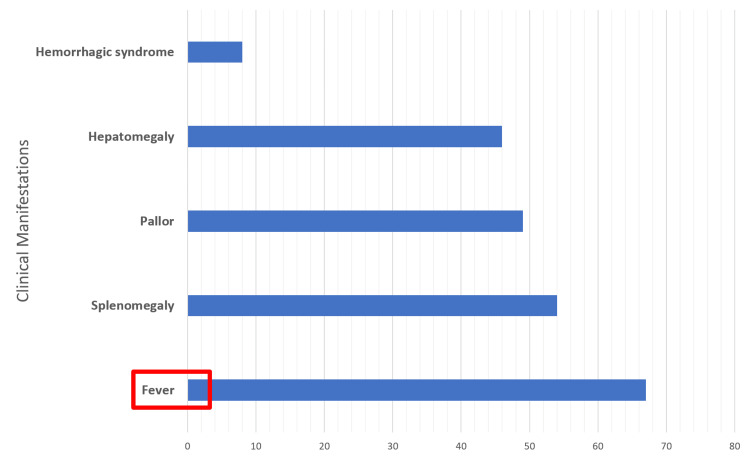
Prevalence of clinical symptoms in patients emphasizing the predominance of fever and splenomegaly.

Fever was the most common reason for medical consultation, reported in 67 (96.95%) cases, followed by abdominal distension in 46 (67%) and pallor in 49 (71%) cases. The clinical triad of fever, pallor, and splenomegaly was observed in 54 (78%) patients. Hemorrhagic syndrome, a factor indicating the severity of VL, was noted in eight (11.9%) cases, presenting as epistaxis (Figure 1).

As for the presumptive biological diagnosis, a complete blood count was performed on all patients. It revealed normochromic normocytic nonregenerative anemia with poikilocytosis and anisocytosis in 67 (97.61%) cases of variable severity, including severe anemia with Hb <4 g/dL in two children. This was followed in order of frequency by thrombocytopenia in 61 (87.82%) patients and leukopenia, mainly made up of neutropenia, in 39 (56.35%) cases. Severe thrombocytopenia with platelets <50,000/mm^3 ^was found in 27 (39.73%) cases, and severe leukopenia with a white blood cell count <1,500/mm^3^ was found in nine (12.55%) cases. All disturbances of the blood formula indicated pancytopenia, which was present in 39 (56.34%) patients. The hepatic assessment was disturbed in 33 (47.67%) patients. A biological inflammatory syndrome was observed in 56 (80.95%) patients. No suggestive image of hemophagocytosis was reported.

**Figure 2:**
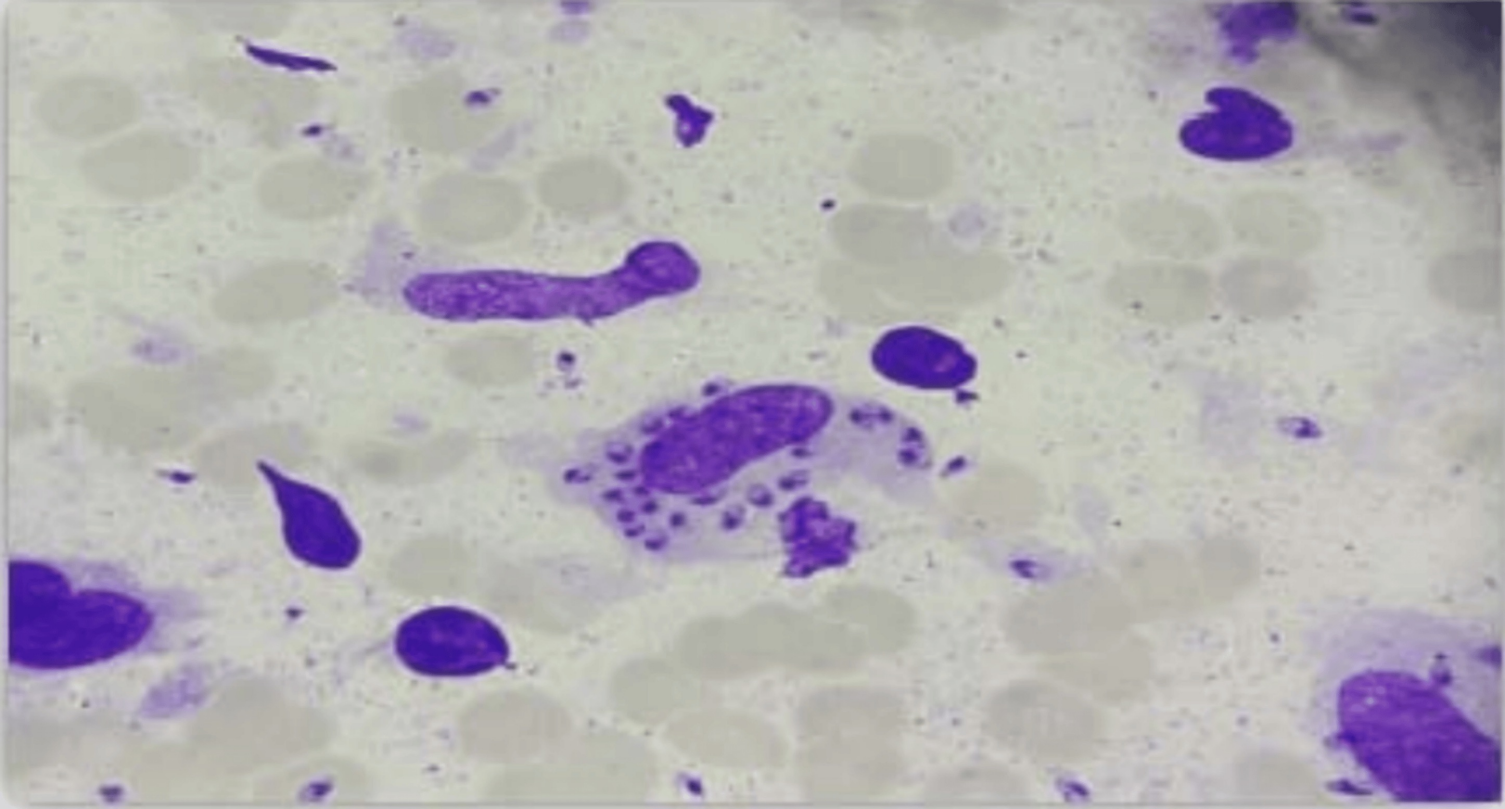
MGG stained bone marrow aspiration showing amastigote forms of Leishmania bodies. MGG: May-Grünwald Giemsa

The diagnosis was confirmed by direct examination of bone marrow stained with MGG, which represents the gold standard laboratory test for VL. In 62 (89.15%) patient samples, the presence of Leishmania bodies was noted (Figure 2). The indirect diagnosis was made using screening techniques such as enzyme-linked immunosorbent assay (ELISA), indirect immunofluorescence (IIF), and rapid diagnostic test associated with the western blot confirmation technique, which allowed for the diagnosis of seven (11%) remaining cases.

Regarding treatment, N-methylglucamine (Glucantime) was used in first place at a dose of 60 mg/kg/day IM, from 21 to 28 days. This molecule was the cause of stibio-intolerance in one patient and of stibio-intoxication (kidney failure in one case and anemia with cytolysis in one other case). The liposomal form of amphotericin B (AMBISOME) was used in six children in cases of relapse, lack of improvement, or intolerance to N-methylglucamine. Unlike Glucantime, AmBisome has reduced renal toxicity. All patients were rigorously monitored to assess clinical improvement and treatment tolerance. A favorable evolution was observed in the majority of patients with an improvement in general condition, regression of hepatosplenomegaly, apyrexia obtained in an average duration of eight days, and a regression of biological disturbances in one to 2.5 months. We deplored three deaths and two relapses.

## Discussion

Infantile visceral leishmaniasis (IVL) is a serious public health problem in Morocco. It has experienced an evolution of endemicity over time, with an incidence increase in the early 2000s. A decline in cases was observed in recent years as a result of the preventive action taken by health authorities.

In this study, the average age was two years and 11 months with predominance for infants and toddlers. This elective affinity for preschool children is explained by a range of arguments, including the immaturity of the child's immune system. It is also favored by frequent contact with dogs and young children. We should also point out that some authors describe a more marked affinity of sandflies for young children, which can explain the rarity of this infection in older children [6].

Male children appear statistically more affected by the disease (53%). Other Moroccan and non-Moroccan studies report the same male predominance [6-8]. The probable explanation in our context is that, in Moroccan culture, especially in rural areas, girls are more likely to wear long, covering clothes that protect them from sandfly bites.

Analysis of the geographical origin of affected children confirms that there is a distribution of kala-azar in Morocco along the following three lines: a main axis represented by the foci of the far north; the second, parallel to the first, which bends to end in Marrakesh; and the third axis, less important, located at the foot of the High Atlas, including the foci of Irherm and Errachidia [6]. All these territories offer identical bioclimatic conditions, an arid or semi-arid character favorable to the development of leishmaniasis visceral (LVI). Furthermore, nearly 80% of the children came from rural or semi-urban regions. These results, similar to other studies, suggest that poor socioeconomic conditions as well as lack of hygiene contribute to the sustainability of this parasitosis [4,9].

Clinically, the characteristic triad (pallor, fever, and splenomegaly) has been objectified in most studies. None of the IVL signs are pathognomonic. Indeed, several conditions can mimic its initial clinical presentation; this has been reported in all pediatric series with a frequency that varied from 71 to 100%[9-11]. Fever is most often moderate, resistant to antibiotics, oscillating with shivers mimicking a septicemic picture, occurring in waves of several days or weeks interspersed with periods of apyrexia even in the absence of any treatment. It is known as "the mad fever." In our series, 97% of the children were febrile at the time of the consultation. The apyrexia on admission in one patient could be due to the anarchic character of the fever. The pallor, meanwhile, varies from 50% in Idrissi et al. (Fez - Morocco) to 100% in Lito et al. (Albania) [7,12,13]. This can be explained by the subjective nature of this symptom which remains variable according to the skin color of the patient and the examiner's estimation.

In IVL, splenomegaly is an early and frequent sign. The spleen is usually smooth, painless, firm, and mobile distorting the abdomen. Hepatomegaly (HMG) is particularly common, discreet or moderate, and rarely voluminous. The presence of lymphadenopathy during VL has been reported too in the majority of series and its incidence varies from 8 to 36%. A hemorrhagic syndrome can also be seen in VL. It affects 8 to 30% of cases in pediatric series (Table 1).

**Table 1:** Comparison of different clinical symptoms according to literature.

Studies	Fever	Pallor	Splenomegaly	Hepatomegaly	Adenopathy	Hemorrhagic syndrome
Zougaghi et al. [6]	92.4%	76%	98.9%	48.4%	-	-
Idrissi et al. [7]	94.5%	50%	97.7%	47.4%	9%	13.7%
Zait et al. [8]	77.4%	43.6%	83%	57.7%	9.8%	11.2%
Safi et al. [9]	70%	70%	70%	70%	30%	-
Minodier and Garnier [11]	90%	64%	100%	63%	36%	-
Lito et al. [12]	100%	100%	100%	100%	8%	8%
Totan et al. [13]	100%	75%	100%	85%	25%	30%
Aissi et al. [14]	79.9%	-	97.9%	47.3%	-	-
Aboudourib [15]	95.16%	93.54%	96.7%	43.54%	6.54%	32.2%
Elghaidi [16]	97.5%	95.05%	92.50%	39.50%	5.23%	-
Present study	97.6%	71.4%	90%	64%	21%	11.9%

The determinism of the clinical manifestations remains partially unclear, it would depend on the patient's age and their immune status or genetics. A human-parasite contact therefore does not necessarily lead to a leishmaniasis disease.

Anemia is extremely common. It is generally normochromic, normocytic, and nonregenerative, initially moderate then gradually worsens during the evolution to reach, in extreme cases, results rates of hemoglobin less than 4 g/dL if not treated. It results from two different mechanisms, the first is central, due to dyserythropoiesis by irritation of the bone marrow in contact with parasite antigens; the second mechanism is due to hypersplenism in one hand, and to the activation of complement by the formation of antigen-antibody complexes in the other hand [17].

Leukopenia was present in 57% of patients. Hypersplenism is once again implicated in the peripheral destruction of these cells and exposes the patient to numerous infectious complications that can worsen the prognosis and complicate the management of the infection. Thrombocytopenia is present in approximately 70-90% of IVL cases, depending on the series, and may be responsible for severe hemorrhagic syndromes requiring repeated transfusions. Similar blood cell count results have been reported in different series [7,8,18]. The erythrocyte sedimentation rate is a good element of orientation. Its acceleration was mentioned in several pediatric series [7,19]. In this study, this parameter was not systematically requested. The C-reactive protein was positive in 80.95% of patients reflecting the inflammatory syndrome. Bone marrow aspiration is the gold standard technique for the diagnosis of IVL. It confirms the diagnosis in 72-100% of patients [6,9,11,12]. This test may be falsely negative due to poor handling of the smear or operator’s error.

For immunocompetent patients, IVL generates a humoral immune response that is strong enough to be explored. The available techniques are numerous, they use antigenic preparations containing either whole parasites, obtained from cultured promastigotes, or parasite extracts. In our study, the patients benefited from screening tests - ELISA, IFI, and rapid diagnostic test - associated with the western blot test as a confirmation technique. The performance of serology varies according to the technique and the antigens used, as well as the specific epidemiology of each country. Hence, there is an interest in mastering the epidemiological specificities of our country to select the appropriate reagents and techniques. Western blot remains the gold standard confirmation method offering the greatest sensitivity and specificity, but its expensive cost limits its use in our context [20-22]. It is highly desirable to standardize PCR performed on total peripheral blood collected from the elbow crease to avoid bone marrow aspiration, which remains an invasive procedure, especially for children.

IVL is a chronic, slowly progressive disease that can last several months or even years. Without treatment, the evolution is marked by the aggravation of clinical symptoms and biological signs leading to a cachexic state. Death occurs most often by intercurrent infections or hemorrhage. When treatment is instituted early enough, the evolution is favorable and leads to a clinical cure. Conventional treatment uses pentavalent antimonials at a dose of 60 mg/kg/day IM. Amphotericin B is used as a second relay in case of no improvement or intolerance to N-methylglucamine [6].

The study's limitations include its retrospective design, which may introduce recall and selection bias, and the exclusion of incomplete patient records, potentially affecting the comprehensiveness of the findings. Additionally, the study's focus on a single geographic region may limit the generalizability of the results, especially since the region in the south of Morocco, which is the focus of this study, is not endemic for IVL, unlike the northern region, where the disease is more prevalent. Future research should employ prospective designs, larger sample sizes, and broader demographic representation to provide a more comprehensive understanding of IVL patterns and treatment challenges.

## Conclusions

Infantile visceral leishmaniasis is a condition that seems to be re-emerging in Morocco, especially in children. This study highlights the epidemiological, clinical, and diagnostic features of infantile visceral leishmaniasis (IVL) in Morocco, with an emphasis on the importance of early diagnosis and treatment for better outcomes. The findings suggest that IVL predominantly affects infants and toddlers, with a slight male predominance, and is often seen in rural and low-income areas. Fever, abdominal distension, pallor, and splenomegaly are the most common clinical symptoms, with normocytic normochromic anemia being the most frequent laboratory finding. Bone marrow aspiration remains the gold standard for diagnosis, confirming the presence of leishmania bodies in most cases. Serological techniques like ELISA and western blot are valuable adjuncts. Despite the availability of effective treatments like N-methylglucamine, the study found some cases of drug intolerance and relapses, underscoring the need for careful monitoring. The study also highlights the importance of public health interventions and preventive measures to reduce VL transmission, particularly in high-risk rural areas. An early diagnosis is necessary for the improvement of the prognosis and depends on a better knowledge of the epidemiological-clinical and biological profile of the patient.
